# Copper Intrauterine Device Migration Into Cesarean Scar Niches Without Complications: A Case Report

**DOI:** 10.1155/crog/4724648

**Published:** 2025-12-20

**Authors:** Zahra Salehi, Roya Derakhshan, Neda Hashemi, Fateme Kourkinejat, Samaneh Rokhgireh

**Affiliations:** ^1^ Endometriosis Research Center, Iran University of Medical Sciences, Tehran, Iran, iums.ac.ir; ^2^ Department of Obstetrics and Gynecology, School of Medicine, Iran University of Medical Sciences, Tehran, Iran, iums.ac.ir

**Keywords:** cesarean scar niche, cesarean section, hysteroscopy, intrauterine device, IUD migration

## Abstract

**Introduction:**

Intrauterine devices (IUDs), both hormonal and nonhormonal, are widely used contraceptive methods, with approximately 20% of reproductive‐aged women worldwide utilizing them. Although IUDs are generally considered safe and effective, complications such as displacement and migration can occur. We report a rare case of copper IUD migration into a cesarean section scar niche.

**Case Presentation:**

A 28‐year‐old woman presented to the emergency department with complaints of lower abdominal pain and abnormal vaginal bleeding. She had received a T‐shaped copper IUD 2 years prior. Transvaginal ultrasound revealed an inverted IUD within the endometrial cavity, with one arm embedded in the cesarean scar defect. The device was successfully removed via hysteroscopy without complication.

**Conclusion:**

This case highlights the importance of imaging evaluation in symptomatic IUD users with a history of cesarean delivery.

## 1. Introduction

Numerous contraceptive methods are currently available, among which hormonal and nonhormonal intrauterine devices (IUDs) are widely used and popular options [1]. The use of IUDs to prevent pregnancy dates back to the early 20th century. Today, two main types are available: copper‐based and progestin‐releasing IUDs, both of which are considered effective, safe, and acceptable long‐term contraceptive methods [2]. The global use of IUDs has markedly increased over the past 30 years, with approximately 20% of women of reproductive age using these devices worldwide [3]. Although IUDs are generally safe, complications can occasionally occur, and their incidence has risen in parallel with increased utilization [4]. One of the most significant complications is device displacement, often first indicated by the inability to visualize the retrieval threads. This can lead to adverse outcomes such as ectopic pregnancy, device expulsion, uterine perforation, partial or complete migration into the myometrium, or even migration into adjacent pelvic or abdominal organs [5]. Common sites of extrauterine migration include the omentum, rectum, sigmoid colon, peritoneal cavity, and urinary bladder [6]. However, migration of an IUD into a cesarean section scar or scar niche is exceedingly rare and underreported [7]. Herein, we present a case of copper IUD migration into a cesarean scar niche in a symptomatic woman.

## 2. Case Presentation

A 28‐year‐old woman, Gravida 2 Para 2, with a history of two cesarean deliveries, presented to the emergency department with complaints of lower abdominal pain and abnormal vaginal bleeding. The abnormal bleeding had persisted for the past 8 months, while the lower abdominal pain had worsened the night before admission. She also reported urinary frequency and dysuria. Her last menstrual period had occurred 20 days prior.

The patient had undergone her first cesarean section due to a twin pregnancy. A T‐shaped copper IUD had been inserted 2 years earlier as a method of contraception. She had not attended routine follow‐up after insertion and only presented for evaluation 1 month prior to admission, at which time the IUD retrieval thread was not visualized. A Pap smear performed during that visit was normal, but she did not return for further assessment.

On examination, the patient′s vital signs were stable. Abdominal palpation revealed tenderness in the hypogastric region without signs of guarding. On speculum examination, the cervix appeared normal and nulliparous; however, the IUD thread was not visible. The patient reported persistent spotting. Laboratory tests revealed a hemoglobin level of 11.2 g/dL, a white blood cell count of 13,200/*μ*L, and neutrophils comprising 78% of the total leukocytes. The urine pregnancy test was negative.

Transvaginal ultrasound demonstrated a uterus measuring 40 × 51 × 75 mm. The IUD was seen inverted within the endometrial cavity, with one horizontal arm embedded in the anterior uterine wall at the level of the cesarean section scar. The endometrial cavity contained a small amount of fluid, and the endometrial thickness was measured at 2 mm. Both ovaries appeared normal.

Pelvic computed tomography (CT) confirmed sonographic findings: (a) sagittal CT view showing the T‐shaped copper IUD inverted within the endometrial cavity and (b) axial CT view demonstrating the arm of the IUD embedded into the anterior myometrial defect consistent with a cesarean scar niche; note the intact serosal contour without evidence of free intraperitoneal device migration (Figure 1).

Figure 1(a, b) Computed tomography showed dislocated IUD in the niche.

**Figure:**
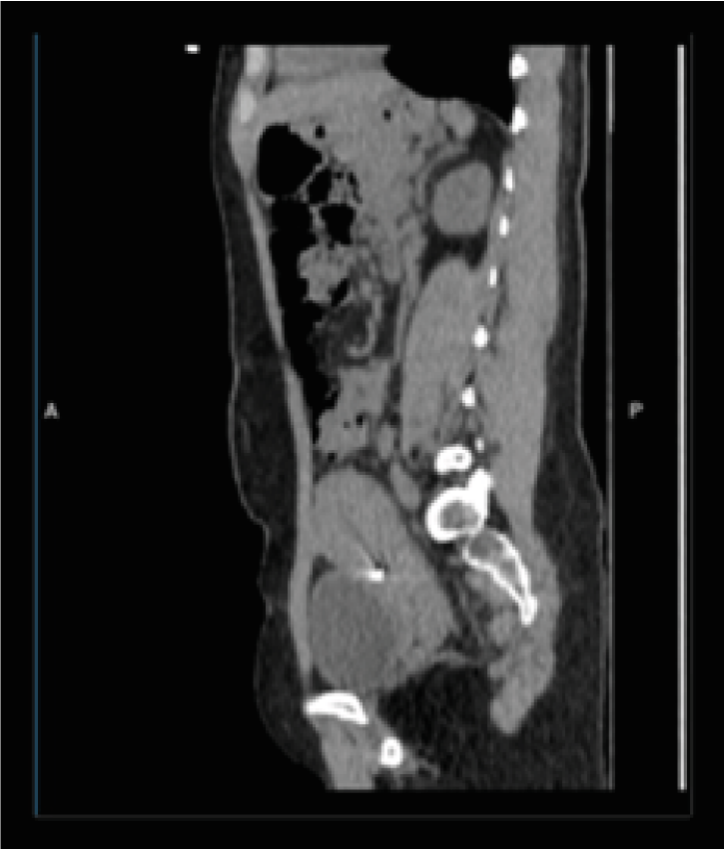
(a)

**Figure:**
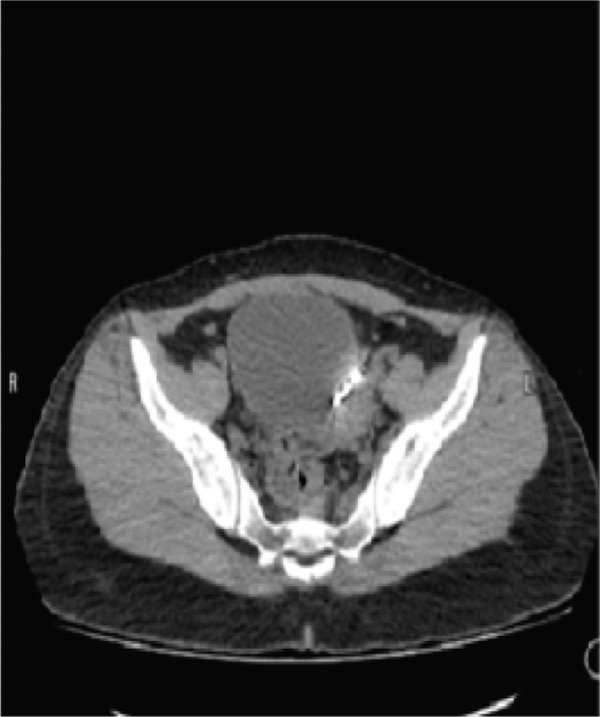
(b)

Given these findings, the patient was taken to the operating room for hysteroscopic evaluation under general anesthesia. Hysteroscopy revealed a normal endometrial cavity and bilateral tubal ostia.

Intraoperative hysteroscopic images revealed (b) a view of the inverted IUD within the endometrial cavity with the retrieval thread directed toward the fundus. (a) Closer view demonstrated the device arm penetrating into the anterior cesarean scar niche. The niche appears as a shallow mucosal defect lined by fibromuscular tissue with prominent vascularity (Figure 2). The device was gently grasped and successfully removed. Further inspection revealed that the niche was located anteriorly at the site of the previous cesarean incision.

Figure 2(a, b) IUD located in the niche.

**Figure:**
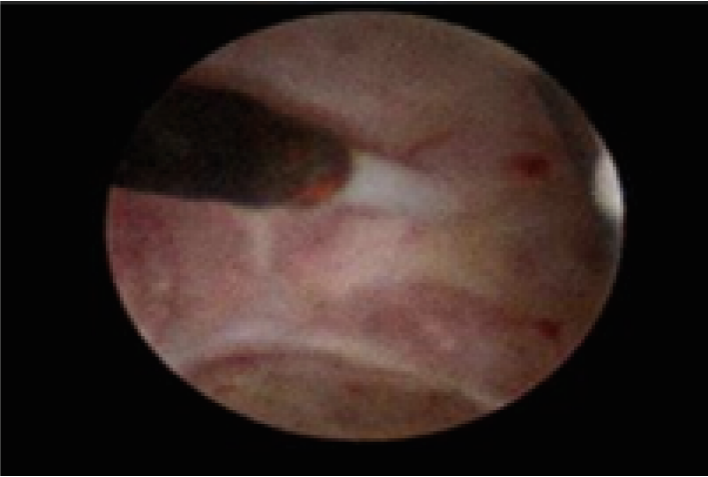
(a)

**Figure:**
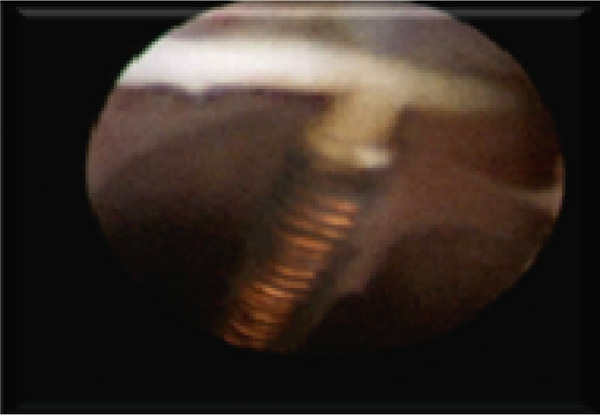
(b)

As the patient expressed no desire for future fertility, the niche was ablated due to her history of prolonged abnormal bleeding and the intraoperative finding of fibromuscular stroma with prominent blood vessels at the niche site. Postoperatively, the patient′s abdominal pain resolved, and she was discharged in good general condition on the same day.

## 3. Literature Review

A defect at the site of a previous cesarean section can lead to the formation of a uterine scar niche, which may result in postmenstrual uterine bleeding. When the residual myometrial thickness is reduced, the risk of uterine rupture in late pregnancy increases. Additionally, such defects can predispose to abnormal implantation of the gestational sac or placenta within the scar area. IUDs are a highly effective form of contraception. Levonorgestrel‐releasing intrauterine devices (LNG‐IUDs) have the added benefit of reducing menstrual bleeding. However, in women with uterine scar defects, there is a risk of IUD migration into the cesarean scar niche or diverticulum [8]. Although this complication is rare, several similar cases have been described in the literature (Table 1). Verest et al. [7] reported a 38‐year‐old woman with a history of three cesarean deliveries who presented with severe lower abdominal pain following intercourse. She had an LNG‐IUD inserted 2 years after her last cesarean, which was initially confirmed to be correctly placed. Then, 3 years later, transvaginal ultrasound revealed that the IUD had migrated into a cesarean scar niche, with fluid accumulation in the uterine cavity. Hysteroscopy confirmed the IUD′s presence within the isthmocele, and the device was removed. The patient′s symptoms resolved, and no surgical repair of the niche was needed.

**Table 1:** Summary of studies with similar reports with our study.

**Author**	**Age of patient**	**Location of IUD displacement**	**History of cesarean section**	**Presence of symptoms**	**Date IUD placed**	**Important factors**
Veset et al.	38 years	Caesarean scar niche (isthmocoele)	Three cesarean sections	Lower abdominal pain, amenorrhea, diffuse abdominal tenderness	2 years after last cesarean	Ultrasound, hysteroscopy, niche repair not necessary, oral contraceptive postremoval

Jing et al.	34 years	Between bladder and abdominal wall, penetrating musculus rectus abdominis	One cesarean section	Ruptured abdominal wall abscess, intermittent abdominal pain	17 years after cesarean, 5 years after IUD insertion	Ultrasound, pelvic CT scan, surgical removal, delayed symptoms

Park et al.	41 years	Cesarean scar	Three cesarean sections	Menstrual irregularities, pelvic pain	Not specified	Transvaginal ultrasound, displacement recurrence

In another case, Jing et al. described a 34‐year‐old woman who presented with a ruptured abdominal wall abscess. She had undergone a cesarean section 17 years earlier and had a copper IUD inserted 5 years prior to presentation. She had experienced intermittent abdominal pain for 4 years. Imaging revealed that the IUD had migrated through the cesarean scar into the rectus muscle, with one arm of the device penetrating the abdominal wall. Surgical removal confirmed the diagnosis, and the patient recovered uneventfully. This case highlights the potential for significant complications from migrated IUDs, even in the absence of acute symptoms for several years.

Park and King [9] reported a 41‐year‐old woman with a history of three cesarean deliveries who underwent routine IUD removal and reinsertion. A follow‐up ultrasound revealed that the newly inserted IUD had migrated into the cesarean scar niche. Initially asymptomatic, the patient later developed menstrual irregularities and pelvic pain, prompting removal and replacement of the device. Notably, the replacement IUD was also found to be displaced into the scar niche shortly after insertion.

These cases underscore that cesarean scar niches may serve as sites of IUD migration and retention. In our case, the patient presented with abnormal uterine bleeding and lower abdominal pain, which prompted further imaging. Transvaginal ultrasound was critical in identifying the malpositioned device and guiding management.

Given the risk of asymptomatic displacement, particularly in women with a history of cesarean delivery, regular follow‐up and imaging should be considered after IUD insertion. Early identification and removal of a displaced IUD are essential to prevent complications.

## 4. Discussion

IUDs are generally considered safe and effective methods of contraception. However, complications, though rare, can occur. These may include pelvic pain, abnormal uterine bleeding, spotting, chronic pelvic inflammatory disease, and an increased risk of unplanned or ectopic pregnancies [10]. Spontaneous migration of the IUD into extrauterine structures is sporadic but can result in serious complications such as peritonitis, appendicitis, intestinal obstruction, vesicouterine fistula, or even sepsis‐related death [11]. When an IUD retrieval thread is not visible during self‐examination or clinical evaluation, several possibilities should be considered. These include: [1] the IUD remains intrauterine but the thread has coiled or detached, or [2] the IUD has perforated the uterine wall and migrated into the abdominal cavity [10]. Risk factors for IUD displacement or migration include a small or irregularly shaped uterine cavity, strong uterine contractions (especially during menstruation), postpartum insertion, and improper insertion technique [1]. An important clinical question arises in asymptomatic patients with incidentally discovered IUD displacement: Should the device be removed? Although the management of asymptomatic cases remains controversial, migration may compromise contraceptive efficacy and carry a risk of delayed complications, making removal a reasonable consideration in many cases [12, 13]. In our patient, the presentation of abnormal uterine bleeding, lower abdominal pain, and urinary symptoms raised the suspicion of IUD displacement. The inability to visualize the retrieval thread during vaginal examination further supported this possibility. Transvaginal ultrasonography is the first‐line and most effective diagnostic tool for evaluating suspected IUD displacement. In some cases, plain abdominal radiographs or CT scans may aid in identifying the location of the device [13]. In this case, transvaginal ultrasound revealed an inverted IUD partially embedded in the anterior uterine wall, with one arm lodged in the cesarean section scar niche. Given the acute clinical symptoms and confirmed malposition, hysteroscopic removal was indicated. Hysteroscopy revealed a well‐formed niche covered by fibromuscular tissue and allowed for safe extraction of the device.

Cesarean scar defects or uterine niches are relatively common following one or more cesarean sections. These defects are often associated with symptoms such as postmenstrual spotting, pelvic pain, and dyspareunia [14]. A previous report documented IUD migration through a scar niche and penetration into the urinary bladder [11]. Emphasizing that these defects can act as sites of pathologic retention or migration.

Regarding the potential association between IUD use and gynecological neoplasms, large epidemiologic studies have not demonstrated a consistent increase in the risk of gynecological cancers with IUDs; in contrast, LNG‐IUDs are associated with decreased endometrial hyperplasia and even therapeutic benefits in selected endometrial hyperplasia/cancer patients [15].

Several case reports and small series have described IUD migration into cesarean scar niches and, rarely, migration into adjacent structures (bladder and abdominal wall). These reports emphasize the variable timing of symptoms, from months to many years after insertion, and the variability of clinical presentation (from incidental imaging findings to abscess or organ penetration). Replacement of a displaced IUD without addressing an underlying niche may be associated with recurrent displacement in some cases, suggesting that identification and consideration of niche management are important when counseling patients.

## 5. Conclusion

Migration of an IUD into a cesarean scar niche is an uncommon but clinically important cause of abnormal bleeding and pelvic pain in women with prior cesarean delivery. We recommend immediate imaging (transvaginal ultrasound ± CT when needed) for symptomatic patients or when retrieval threads are absent, and early hysteroscopic evaluation for diagnosis and safe removal. Close postoperative follow‐up is advised to monitor symptom resolution and to consider niche repair in patients with persistent bleeding or future fertility desire.

## 6. Follow‐Up and Limitations

The patient′s abdominal pain eliminated immediately after the procedure, and she was discharged the same day in good condition. She was advised to return for vaginal bleeding assessment and for clinical review at 6 weeks.

### 6.1. Limitations

Our report is limited by being a single case and lack of limited documentation for long‐term follow‐up and imaging beyond the immediate postoperative period. This limits our ability to report on recurrence or later niche‐related symptoms in this patient. Future reports should include scheduled clinical and imaging follow‐up (for example, at 6 weeks, 3 months, and 6–12 months) to assess symptom resolution and to document any recurrence of device malposition or niche‐related bleeding.

## Ethics Statement

This case report was approved by the local Ethics Committee of the Iran University of Medical Science (IR.IUMS.REC.1402.953). Written informed consent was obtained from the participant.

## Disclosure

All authors have read and approved the manuscript.

## Conflicts of Interest

The authors declare no conflicts of interest.

## Funding

No funding was received for this manuscript.

## Data Availability

The data that support the findings of this study are available on request from the corresponding author. The data are not publicly available due to privacy or ethical restrictions.
